# Simultaneous pharmacokinetic/pharmacodynamic (PKPD) assessment of ampicillin and gentamicin in the treatment of neonatal sepsis

**DOI:** 10.1093/jac/dkab413

**Published:** 2021-11-23

**Authors:** Silke Gastine, Christina Obiero, Zoe Kane, Phoebe Williams, John Readman, Sheila Murunga, Johnstone Thitiri, Sally Ellis, Erika Correia, Borna Nyaoke, Karin Kipper, John van den Anker, Mike Sharland, James A. Berkley, Joseph F. Standing

**Affiliations:** Infection, Immunity and Inflammation, Great Ormond Street Institute of Child Health, University College London, London, UK; KEMRI-Wellcome Trust Research Programme, Kilifi, Kenya; Infection, Immunity and Inflammation, Great Ormond Street Institute of Child Health, University College London, London, UK; Quotient Sciences, Mere Way, Ruddington, Nottingham, UK; KEMRI-Wellcome Trust Research Programme, Kilifi, Kenya; Centre for Tropical Medicine & Global Health, Nuffield Department of Medicine, University of Oxford, Oxford, UK; Infection, Immunity and Inflammation, Great Ormond Street Institute of Child Health, University College London, London, UK; Quotient Sciences, Mere Way, Ruddington, Nottingham, UK; Quotient Sciences, Mere Way, Ruddington, Nottingham, UK; Global Antibiotic Research & Development Partnership (GARDP), Genève, Switzerland; Global Antibiotic Research & Development Partnership (GARDP), Genève, Switzerland; Drugs for Neglected Diseases Initiative (DNDi), Nairobi, Kenya; Institute of Chemistry, University of Tartu, Tartu, Estonia; Department of Paediatric Pharmacology and Pharmacometrics, University Children’s Hospital Basel, University of Basel, Switzerland; Division of Clinical Pharmacology, Children’s National Hospital, Washington, DC, USA; Paediatric Infectious Diseases Research Group, Institute for Infection and Immunity, St. George’s, University of London, London, UK; Quotient Sciences, Mere Way, Ruddington, Nottingham, UK; Centre for Tropical Medicine & Global Health, Nuffield Department of Medicine, University of Oxford, Oxford, UK; The Childhood Acute Illness & Nutrition (CHAIN) Network, Nairobi, Kenya; Infection, Immunity and Inflammation, Great Ormond Street Institute of Child Health, University College London, London, UK; Pharmacy Department, Great Ormond Street Hospital for Children, NHS Foundation Trust, London, UK

## Abstract

**Objectives:**

This study aimed to simultaneously investigate the pharmacokinetics of ampicillin and gentamicin, currently the WHO standard of care for treating neonatal sepsis.

**Methods:**

Pharmacokinetic data were collected in 59 neonates receiving ampicillin and gentamicin for suspected or proven sepsis in the NeoFosfo trial (NCT03453177). A panel of 23 clinical *Escherichia coli* isolates from neonates with sepsis, resistant to either ampicillin, gentamicin or both, were tested for susceptibility using chequerboards. Pharmacokinetic/pharmacodynamic (PKPD) modelling and simulations were used to compare single-agent (EUCAST MIC) and combination (chequerboard MIC) target attainment with standard dosing regimens.

**Results:**

A model was established that simultaneously estimated parameters of a one-compartment ampicillin model and a two-compartment gentamicin model. A common clearance for both drugs was used (6.89 L/h/70 kg) relating to glomerular filtration (CL_GFR_), with an additional clearance term added for ampicillin (5.3 L/h/70 kg). Covariate modelling included *a priori* allometric weight and post-menstrual age scaling of clearance. Further covariate relationships on renal clearance were postnatal age and serum creatinine.

Simulation-based PKPD assessments suggest good Gram-positive (MIC ≤ 0.25 mg/L) cover. However, less than one-quarter of neonates were predicted to receive efficacious coverage against Enterobacterales (MIC ≤ 2 mg/L). The benefit of the ampicillin/gentamicin combination was limited, with only 2/23 *E. coli* clinical strains showing FIC index < 0.5 (synergy) and most in the range 0.5–1 (suggesting additivity). Simulations showed that feasible dosing strategies would be insufficient to cover resistant strains.

**Conclusions:**

PKPD simulations showed ampicillin and gentamicin combination therapy was insufficient to cover Enterobacterales, suggesting the need for alternative empirical treatment options for neonatal sepsis.

## Introduction

In neonatal care, infectious diseases associated with antimicrobial resistance (AMR) are of increasing concern and need to be addressed on a global scale. Infection is one of the major causes of neonatal deaths worldwide, with Asia and sub-Saharan Africa carrying the largest burden.^1^ Microbial patterns vary globally. In high-income countries, primary strains are *Escherichia coli* and group B Streptococcus (GBS, *Streptococcus agalactiae*),^2–4^ but in low- and middle-income countries (LMICs) they are *Klebsiella* spp., *E. coli* and *Staphylococcus aureus*.

The WHO’s current recommended treatment for neonatal sepsis is a narrow-spectrum β-lactam agent in combination with gentamicin.^5^^,^^6^ Combination of gentamicin with ampicillin has an acceptable safety profile, but the limited access to therapeutic drug monitoring to monitor gentamicin exposure can be seen as a major safety concern in LMICs. Although resistance to ampicillin and gentamicin has been shown,^7^^,^^8^ in particular for Gram-negative clinical isolates in Asia and Africa, this combination therapy remains a vital part of standard-of-care (SOC) neonatal antibiotic therapy.^4^^,^^9^

Both drugs have previously been studied as monotherapy.^10–12^ Pharmacokinetics/pharmacodynamics (PKPD) of the combination regimen and the degree to which the PK of both drugs are correlated within a patient and whether target attainment may differ when accounting for both agents together, however, has not been described so far, to the best of our knowledge. We therefore analysed ampicillin and gentamicin plasma concentrations from the PK samples taken in the recent NeoFosfo study,^13^ where both drugs were given as SOC treatment alongside fosfomycin. A PKPD model was established and evaluated with microbiological synergy data from clinical strains.

## Methods

### Study and drug details

An open-label randomized controlled trial was conducted to assess the safety and PK parameters of IV followed by oral fosfomycin.^13^ In this trial, fosfomycin was administered together with SOC antibiotics (ampicillin plus gentamicin) and compared with SOC alone among hospitalized neonates with clinical sepsis. The NeoFosfo trial was conducted at Kilifi County Hospital (KCH) between March 2018 and March 2019.

Ampicillin (50 mg/kg, administered 12 hourly for participants younger than 7 days and 8 hourly if more than 7 days old), plus 24 hourly gentamicin (3 mg/kg, for participants less than 2 kg, or 5 mg/kg if more than 2 kg) were prescribed as SOC antibiotics at admission.

### PK sampling

Patients allocated to the SOC plus fosfomycin (SOC-F) arm were randomly allocated to one of three early (5, 30 or 60 min) and one of three late timepoints (2, 4 or 8 h) for PK sample collection after the first parenteral and the first oral fosfomycin dose. A fifth sample was collected after the final dose of oral fosfomycin for participants still hospitalized on Day 7. Sample processing and the analytical assay are described in the Supplementary methods, available as Supplementary data at *JAC* Online.

### Data analysis and software

Data pre-analysis and graphical output was created in R (version 3.6.1; R foundation for statistical computing, Vienna, Austria). Model building was performed using NONMEM (Version 7.4; ICON Development Solutions, Ellicott City, MD, USA) with the FOCE + I estimation method. Visual predictive checks (VPCs) were performed with Perl-speaks-NONMEM (PsN, version 4.8.1). Graphical output was created in R.

### PK model building

For each individual drug, one- and two-compartment structural models were assessed. Inter-individual variability (IIV) was assumed to follow a log-normal distribution and was tested on all parameters. An additive, a proportional and a combined error model were tested. For nested models, a decrease in −2 times the log likelihood of >6.64 was needed to be significant at a level of *P* < 0.01 and >3.84 at a level of *P* < 0.05, for a change in 1 degree of freedom using the chi-squared distribution, respectively.

Allometric scaling of body weight was included using a fixed exponent of 0.75 on clearance terms and linear scaling on volume terms.^14^ To compare parameter estimates with other paediatric and adult studies, parameters were scaled to a standard weight of 70 kg. A previously published neonatal renal maturation function by Rhodin *et al.*^15^ was added to clearance *a priori*.^10^^,^^16^

Further renal clearance maturation after birth, regardless of gestational age, which occurs over the first few days/weeks of life^10^ was implemented. This effect on clearance was detected by Kane *et al*.^13^ through analysis of the fosfomycin data for this study (Equation 1) and was thus also tested in the ampicillin and gentamicin model.

(1) $${\mathrm{PNA}}_{\mathrm{function}}=\theta_{M}+\left(1-\theta_{M}\right)\times \left(1-e^{-PNA_{i}\times \theta_{N}}\right)$$

where PNA_function_ is the introduced covariate relationship, θ_M_ is the population value relating to PNA maturation, PNA_i_ is the individual’s postnatal age and θ_N_ is the population value of the shape parameter. The ability of serum creatinine concentration (SCR) to predict individual variability in renal clearance was tested according to Equation 2, where θ_SCr_ is the population value of the detected creatinine effect and SCR_i_ is the individual’s creatinine measurement.

(2) $${\mathrm{SCR}}_{\mathrm{function}}={\frac{SCR_{i}}{\mathrm{TSCR}}}^{\theta_{\mathrm{SCr}}}$$

The impact of deviation from age-related standard was tested on renal clearance (Equations 1 and 2). The measured SCR was standardized to typical serum creatinine concentrations (TSCR) for the respective age calculated according to Ceriotti *et al*. (Equation 3).^17^
 

(3) $$\mathrm{TSCR}\left(\mu \mathrm{mol}\right)=-2.37330-12.91367\times \ln({\mathrm{PNA}}_{\mathrm{years}})+23.93581\times {\left({\mathrm{PNA}}_{\mathrm{years}}\right)}^{0.5}$$

### Model evaluation

Goodness-of-fit (GOF) plots and VPCs served as tools to evaluate improvement in model appropriateness and predictivity throughout model development steps and to assess the final model. A non-parametric bootstrap (*n* = 1000) was performed on the final model to test parameter precision and robustness. Perl-speaks-NONMEM (PsN) was used in the bootstrap analysis and VPCs.

### Combination antibiotic testing

Clinical *E. coli* strains collected from neonates with sepsis^18^ from centres with high prevalence of ESBL-producing Enterobacteriaceae were analysed for antibiotic susceptibility when challenged with ampicillin, gentamicin and the combination of both drugs. The EUCAST disc diffusion test,^19^ with reference to EUCAST clinical MIC breakpoints,^20^ was used to determine the susceptibility of each strain to each drug.

MICs for each strain were determined by broth microdilution methods in accordance with BSAC guidance.^19^ MICs were determined for the single drugs ampicillin and gentamicin, as well as for the combination of both drugs through synergy testing. Synergistic relationships were tested against single *E. coli* isolates using a 96-well plate standard 2D chequerboard assay.^20^ The FIC index (FICI) was calculated and reported as synergistic when <0.5, additive when within the range of 0.5–2, and antagonistic when >2. The presence of commonly found resistance genes was established by multiplex PCR with previously described primers and amplification conditions.^21^^,^^22^

### PK simulations

Simulations were carried out to explore ampicillin and gentamicin PTA using the registered dose and frequency as stated by the *WHO Pocket Book of Hospital Care for Children.*^5^ Ampicillin was simulated at 50 mg/kg with a frequency of q12h for PNA ≤ 7 days and q8h for PNA > 7 days. Gentamicin was simulated with a single daily dose of 5 mg/kg/day in neonates with PNA ≤ 7 days weighing above 1.5 kg. Gentamicin dose was decreased to 3 mg/kg/day in low-birthweight neonates and increased to 7.5 mg/kg/day in neonates older than 7 days. Both drugs were simulated to be infused over 5 min. As ampicillin dose recommendations vary across formularies^23^ and severity of infection, additional simulations were also carried out with the highest proposed dose (100 mg/kg) using infusion rates of 5 min and, to explore prolonged infusion, also with infusion rates of 1 h, 2 h and continuous infusion.

The simulation population (*n* = 10 000) was created using observed demographics from the present study combined with data from an international multi-centre neonatal observational study (NCT03721302). Simulations were carried out in NONMEM with post-processing in R.

Simulated PD targets were compared with EUCAST breakpoints.^24^ Overall target attainment was studied through graphical evaluation of the fraction of time above MIC at steady-state (%*fT*_>MIC_)^25^ for ampicillin and the maximal concentration to MIC ratio (*C*_max_/MIC) for gentamicin at Day 2 of treatment.^26^ In agreement with common practice, *C*_max_ was defined as the gentamicin concentration 30 min after the end of injection. Simulation results were also compared with results of the antibiotic synergy 2D chequerboard study. PTA was calculated for ampicillin and gentamicin MICs observed in combination with the respective other drug.

### Results

#### Patients and demographics

**Figure 1.**
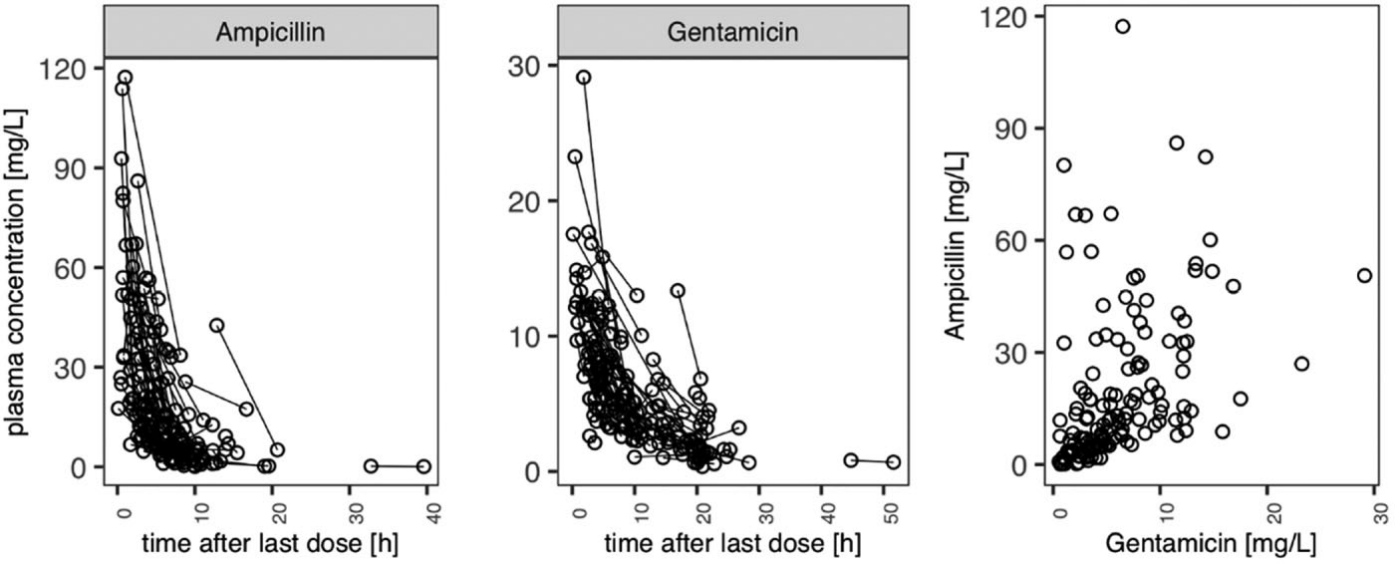
Observed plasma concentration versus time since last dose for ampicillin (left) and gentamicin (middle). Each line represents a single patient’s profile. Correlation of observed ampicillin and gentamicin concentrations is shown on the right.

The final modelling dataset included 373 (181 ampicillin and 192 gentamicin) samples from 59 patients (Figure 1). Demographics and covariate distributions are displayed in Table 1. A total of 34 concentrations were measured below the lower limit of quantification (LLOQ) for ampicillin (0.5 mg/L; *n* = 16) and gentamicin (0.1 mg/L; *n* = 18), respectively.

**Table 1.** Study population demographics

Parameter	SOC arm (*n* = 59)
Age, median (range)	
PNA (days)	1 (0–23)
Gestational age (weeks)	38 (34–44)
Sex, *n* (%)	
Female	24 (41)
Male	35 (59)
Covariates, median (range)	
Weight (g)	2800 (1560–5670)
Creatinine at Day 0 (μmol/L)	96.5 (35–142)
Study design	
Total number of samples, *n*	273
Samples per patient, median (range)	4 (3–5)

#### PK model development

Ampicillin was best described by a one-compartment model with linear elimination when modelled alone. Gentamicin was best described by a two-compartment model with linear elimination when modelled alone. IIV was supported on clearance for ampicillin and clearance and central volume of distribution (*V*_d_) for gentamicin.

Allometric scaling with fixed exponents of 1 and 0.75 for volume and clearance terms, respectively, as well as including the Rhodin^15^ maturation function (Equation 3) on clearance improved each model fit.

The two individual models were subsequently linked in a single model with covariance estimated. To optimize the linked PK model, a common filtration clearance term was estimated for ampicillin and gentamicin, with an additional ampicillin clearance accounting for non-renal pathways, rather than having a separate clearance estimate for each drug (change in objective function value, ΔOFV = −1.5).

Subsequently, the effect of PNA on the combined clearance term, as proposed by Kane *et al*.,^13^ was included using a fixed fraction of clearance at birth and fixed coefficient for clearance maturation (ΔOFV = −32.5). The effect of creatinine in relation to the age-appropriate common creatinine levels according to Ceriotti *et al.*^17^ was estimated as an effect on the combined clearance term and resulted in a significant model improvement (ΔOFV = −7.0; *P* < 0.01).

**Figure 2.**
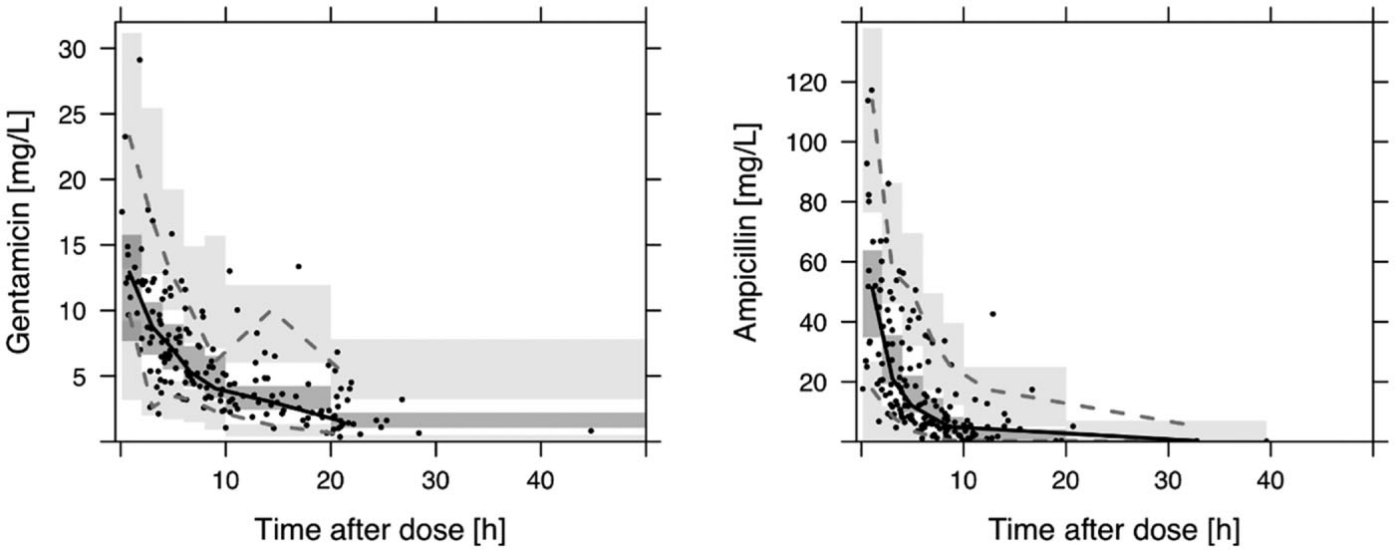
VPC for joint ampicillin and gentamicin model. Black dots, observed values; solid line, median; dashed lines, 5th and 95th percentiles for the observed values; grey areas, 95% prediction interval of respective median and percentiles.

Parameter estimates for the final model along with bootstrap results are shown in Table 2. VPCs for ampicillin and gentamicin from the final combined model are shown in Figure 2.

**Table 2.** Final model parameter estimates

Parameter	Parameter estimates (RSE, %)	IIV (%) (RSE, %)	Bootstrap 95% CI
CL_GFR_ (L/h)	6.89 (8)	38.5 (14)	5.48–8.01
Central *V*d_Gent_ (L)	26.4 (18)	40.6 (40)	9.81–31.2
Q_Gent_ (L/h)	0.956 (28)		0.48–10.3
Peripheral *V*d_Gent_ (L)	6.69 (36)		3.10–16.3
CL_non-GFR_ (L/h)	5.3 (11)	45.4 (16)	4.25–6.54
Central *V*d_Amp_ (L)	52.7 (11)		42.6–65.4
PNA_fract_ (%)	44.9 FIX		
PNA_coeff_	0.117 FIX		
Creatinine on CL_GFR_	−0.423 (53)		−1 to 0.05
Proportional error (%), gentamicin	29.3 (24)		20.6–34
Proportional error (%), ampicillin	53.6 (13)		46.6–60.4

Q, intercompartmental clearance; PNA_fract_, fraction of adult clearance at PNA; PNA_coeff_, coefficient for PNA on clearance; FIX, parameter was fixed and not estimated.

#### Simulation results

**Figure 3.**
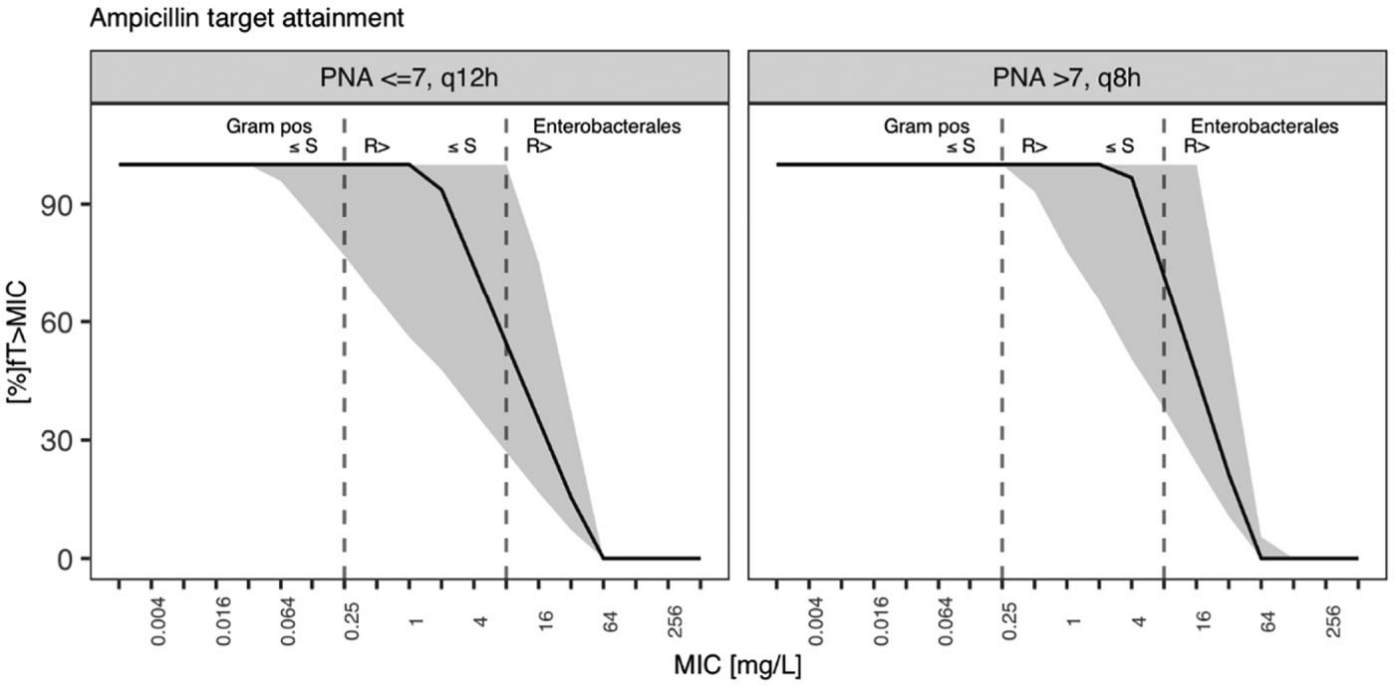
Target attainment as %*fT*_>MIC_ against MICs for simulated ampicillin regimen. Solid line, population median; grey area, 90% prediction interval; dashed lines, EUCAST breakpoints for Enterobacterales and CoNS.

%*fT*_>MIC_ across MIC ranges, including EUCAST breakpoints (Gram-positive strains, MIC ≤ 0.25 mg/L; Enterobacterales, MIC ≤ 2 mg/L) is shown for ampicillin in Figure 3. This was translated into PTA when aiming for 100%*fT*_>MIC_ in Figure S1.

Ampicillin dosing recommendations vary across guidelines and depend on the severity of the infection.^23^ Figure S2 shows the difference in target attainment of the upper and lower end of dose recommendations (50 and 100 mg/kg dosing) when given by 5 min short infusion. Figure S3 shows the scenario of extending the infusion time from 5 min to 1 h and 2 h along with a continuous infusion. Here, only the continuous infusion scenario was able to bring the population median across the Enterobacterales’ MIC, with the green shaded area that represents the 90% prediction interval for the simulated population still not entirely crossing the Enterobacterales MIC for 50 mg/kg. For 100 mg/kg, a continuous infusion scenario was able to cover the entire population.

**Figure 4.**
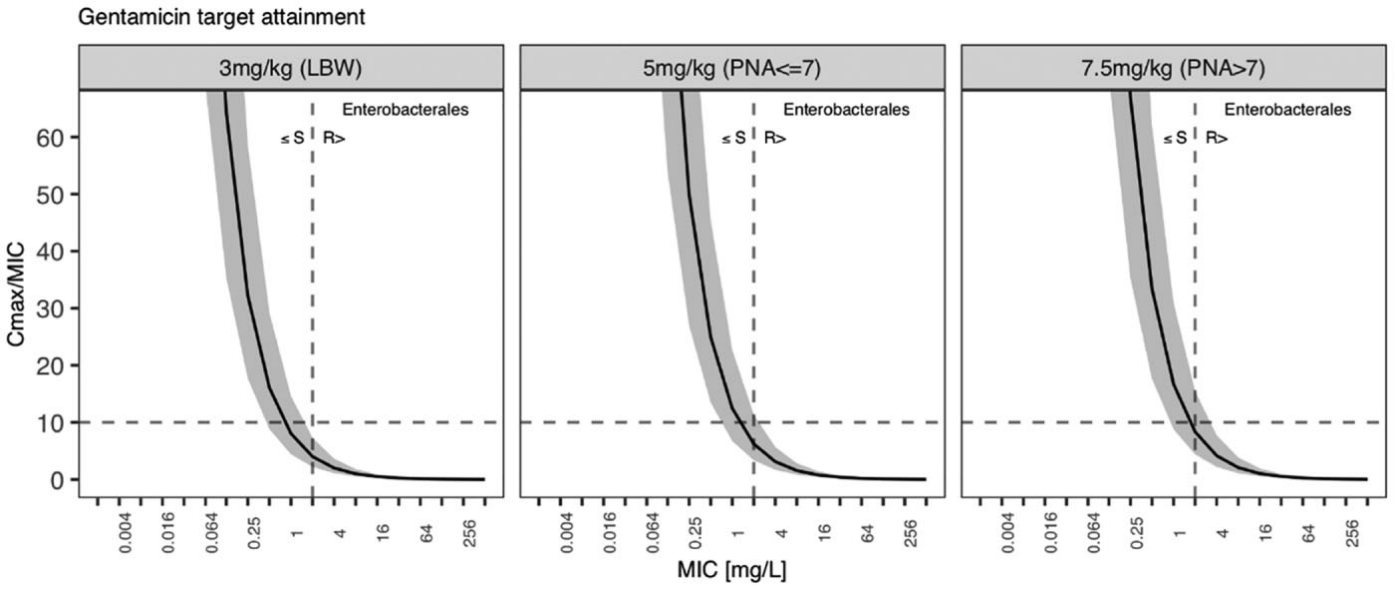
Target attainment as *C*_max_/MIC ratio against MICs for simulated gentamicin regimen. Solid line, population median; grey area, 90% prediction interval; dashed line, EUCAST breakpoint for Enterobacterales.

A *C*_max_/MIC of 10 is regarded to best correlate with aminoglycoside antibacterial efficacy. Target attainment across the MIC range for 2 days of gentamicin therapy is shown in Figure 4, along with the EUCAST breakpoint for Enterobacterales. Figure S4 translates this into a PTA for the simulated population. Both figures show that only a small fraction of low-birthweight neonates is covered against the Enterobacterales EUCAST breakpoint at 2 mg/L MICs. The PTA increased with the increased doses suggested for older and heavier neonates, yet even the 7.5 mg/kg dose in neonates older than 7 days only covers the population median. In contrast, Figure S5 shows the probability of reaching the toxicity target that limits the aminoglycoside therapeutic window. Aminoglycoside toxicity is linked to insufficient drug clearance thus trough concentrations should fall below 1 mg/L before the next dose is administered. Fewer than 25% of the simulated neonates achieve trough concentrations falling below this target during the first days of life. With renal maturation and increasing renal clearance, most of the population, however, show sufficient trough concentrations by 2 months.

#### Combination effect assessment through in vitro 2D chequerboard assay

**Figure 5.**
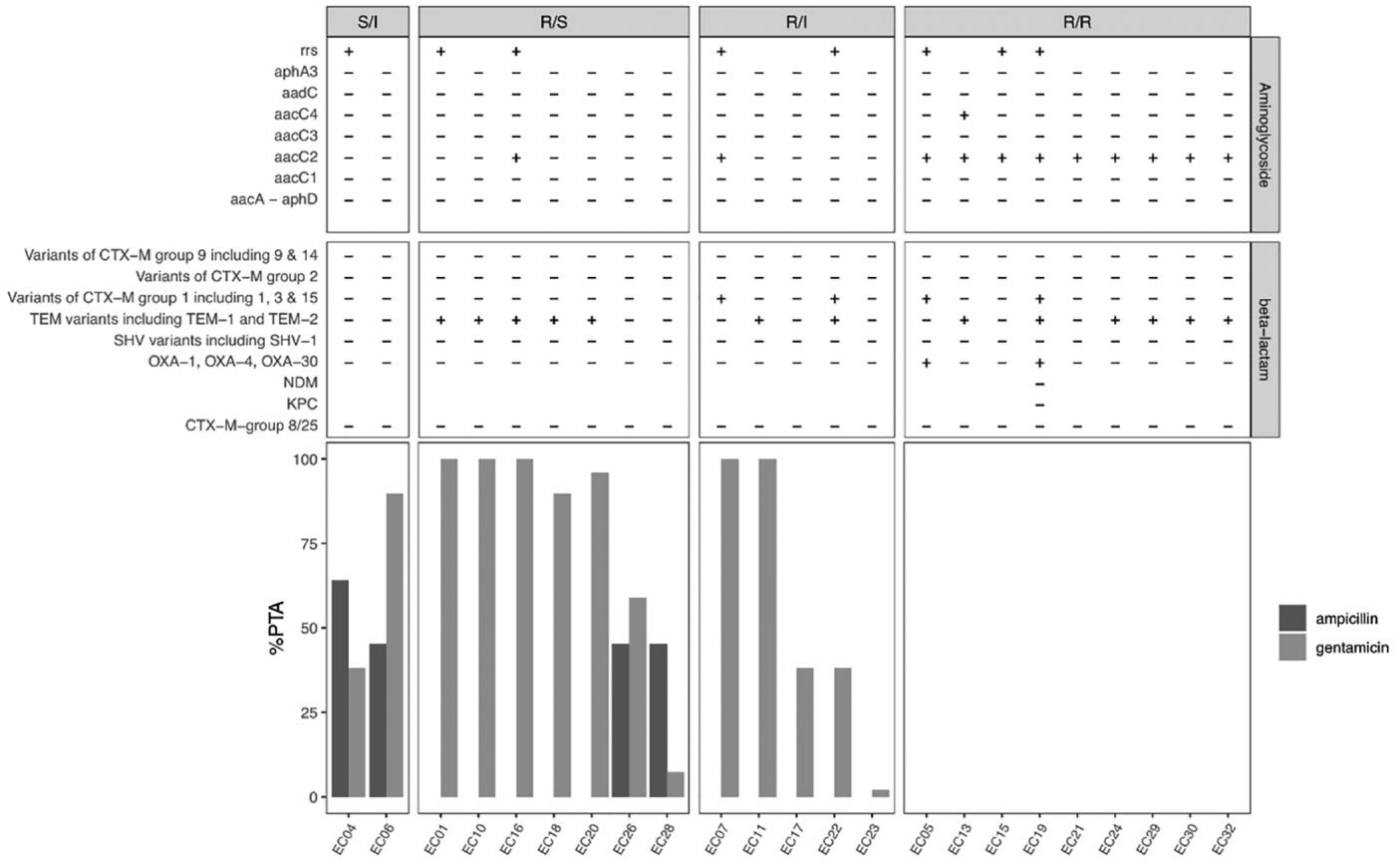
Resistance testing, FICI results and PTA for coverage of MICs for neonatal clinical specimens with the respective combination treatment. Light grey bars, gentamicin; dark grey bars, ampicillin.

Coverage for both organisms in relation to collected *E. coli* MICs from clinical specimens is shown in Figure 5. Ampicillin 100%*fT*_>MIC_ and gentamicin *C*_max_/MIC ratio of >10 were calculated for the isolates’ combined MICs. Sensitive and intermediate-resistant strains were estimated to have partial to full response when treated in this neonatal target population. The magnitude of additive (21/23 strains with FICI between 0.5 and 1) and synergistic effects (2/23 strains with FICI < 0.5) between the combination was insufficient to cover strains resistant to both agents.

## Discussion

We here present a combined ampicillin and gentamicin population pharmacokinetic (popPK) analysis in neonatal sepsis. Our major finding is that the current empirical guidance for neonatal sepsis appropriately manages the most common Gram-positive pathogens responsible for neonatal sepsis with MICs of ≤0.25 mg/L but not common Gram-negative Enterobacterales strains with MICs of ≤2 mg/L. This includes the most frequently detected Gram-positive organism in the LMIC setting, *S. aureus*, which the WHO Pocket Book is targeting (along with GBS and CoNS). These are more likely to be found in infections from vertical transmission, premature neonates or neonates undergoing more invasive treatments. However, Enterobacterales strains, including *Klebsiella* spp. and *E. coli*, are not well covered (Figures 3 and 4).^27–30^ When analysing clinical *E. coli* strains, we observe that despite finding additivity through *in vitro* 2D chequerboard assays for most tested strains (0.5 ≤ FICI ≤ 1) and two strains showing a trend towards synergy (FICI < 0.5) for the combination, this did not bring MICs low enough such that clinically achievable doses would provide adequate coverage (Figure 5).

We used a combined PK modelling approach to assess the common ampicillin/gentamicin SOC regimen’s PK performance in neonates. We found a one-compartment model to best describe ampicillin PK and a two-compartment model to best describe gentamicin PK. Both models were linked after introducing allometry. Elimination of both drugs depends on passive filtration, which is thought to be the main method of clearance for gentamicin, and contributes to approximately half for ampicillin. In the initial combined model the estimated covariance between the sole clearances was around 60%. Therefore, ampicillin clearance was set to the gentamicin clearance (assumed filtration component) plus an estimated non-filtration component. Upon making this assumption, the covariance in the final model between the filtration and non-filtration pathways was estimated to be low at 30%, with the non-filtration component of ampicillin clearance being around 43% of the total, which is similar to previous reports.^31–33^

The assumption that gentamicin is predominantly cleared by glomerular filtration was confirmed by the estimated clearance of 6.89 L/h/70 kg (CL_GFR_) corresponding to a normal adult glomerular filtration rate (GFR) (∼ 115 mL/min). For ampicillin, almost half of the clearance process can be attributed to other pathways.^31–33^ Sjovall *et al.*^32^ determined that 80% of ampicillin can be found unchanged in the urine and that 49% of this excretion can be attributed to tubular secretion. The entire elimination pathway of ampicillin, however, remains unknown in the neonatal population, which is why we describe this with a glomerular filtration-associated term and a second non-filtration term in our model, which accounts for these additional pathways. Maturation functions and the effect of serum creatinine normalized to the age-dependent expected creatinine levels through the function from Ceriotti *et al.*^17^ was subsequently added as a covariate on the filtration-related clearance term only and this resulted in ampicillin clearance estimates close to previously estimated adult values.^11^

Wang *et al.*^33^ have recently evaluated the application of maturation functions to predict neonatal drug clearance, including predictions for ampicillin. They concluded that drugs that are only partially cleared through glomerular filtration are less reliably predicted through these scaling methods. For ampicillin, this meant Wang *et al*.^33^ predicted clearances being twice as high as the observed clearance. In our model, when only applying maturation to the filtration part, overall ampicillin clearance was estimated at 12 L/h/70 kg, which is very much in line with adult ampicillin clearance stated in the summary of product characteristics (11.64 L/h) and observed in adult PK studies (10.7 L/h).^11^ This again highlights the need to assess the feasibility of extrapolation when applying covariate modelling to special population studies, especially when *a priori* effects are implemented. For other β-lactams, the use of generic maturation functions to describe exposure in neonates generally gives good predictions according to Wang *et al.*,^33^ who successfully predicted meropenem clearance in neonates, and a recent study on patients in neonatal, paediatric and adult ICUs showed a common maturation function was applicable to amoxicillin, benzylpenicillin, cefotaxime, meropenem and piperacillin/tazobactam.^34^ It therefore seems that the non-filtration component of ampicillin clearance is close to maturity at birth and implies that higher doses or substitution for amoxicillin should be considered.

The major limitation of our analysis is that the NeoFosfo study’s PK sampling was optimized to assess the plasma concentration trajectories of the primary investigational drug (fosfomycin). Ampicillin and gentamicin treatments were started at different timepoints compared with the investigated fosfomycin therapy, resulting in a broad range of sampling timepoints. This and the fact that more attention is usually paid to the documentation of the investigational drug rather than the SOC drugs given may have led to some uncertainty in this evaluation. We tried to overcome this, however, through data cleaning prior to the analysis and addressing uncertainties with the study site. In addition, whilst the *in vitro* chequerboard analysis was undertaken with organisms isolated from neonates with sepsis and having a range of resistance genes (Figure 5), these isolates came from European hospitals, and hence may not be truly representative of the African setting.

Target attainment simulations with WHO empirical treatment dose guidance for neonatal sepsis^35^ using the final model predicted that Gram-positive coverage throughout the neonatal population would be provided by the regimen’s β-lactam component ampicillin (Figure 3 and Figure S1) when considering a common EUCAST^24^ clinical breakpoint of 0.25 mg/L to reflect susceptible GBS, CoNS and *S. aureus* infections. Current dosing guidance, however, provides poor coverage against common Gram-negative Enterobacterales pathogens. Our analysis reveals that less than one-quarter of neonates treated with this regimen receive efficacious coverage against the gentamicin EUCAST^24^ clinical breakpoint of 2 mg/L (Figure 4 and Figure S4). Increasing gentamicin dosing would need to be accompanied by extending the dose interval beyond the simulated 24 h window, as gentamicin toxicity is linked to insufficient gentamicin clearance and a trough concentration of <1 mg/L is used to guide adequate dose frequency^10^ (Figure S5). During the first week of life the probability of reaching this trough increases, but still leaves more than half the simulated population with a trough level above the threshold. With the restricted access to therapeutic drug monitoring for neonatal care in LMICs, this poses a safety concern in this vulnerable population. Bolus ampicillin dosing showed less than 30% PTA across the simulated population. Adequate ampicillin *fT*_>MIC_ is only reached when applied as continuous infusion along with increasing the total daily dose (Figure S3), which is seldom feasible in the neonatal population and in a global setting.

A neglected topic in antibiotic susceptibility testing is the possibility of synergy. As β-lactamases are proteins and aminoglycosides inhibit protein synthesis, a reasonable hypothesis is that the combination therapy may be synergistic. However, despite FICIs being <1, suggesting at least additivity, only 2/23 tested were <0.5 and so could be classed as synergistic. In Figure 5, where *E. coli* strains extracted from clinical specimens were analysed based on the results of 2D chequerboard synergy testing, the interaction effect was insufficient to restore activity for strains where one or both drugs of the combination were resistant. Even the two strains that showed ampicillin susceptibility and intermediate gentamicin susceptibility were not covered throughout the entire neonatal population. Strains resistant to both drugs (*n* = 8) did not show any coverage, with PTA being <1%. From these results, together with the explored modifications to the dosing regimen, as stated above, the ability to overcome these resistances does not seem feasible with this combination.

Neonatal sepsis due to Gram-negative and MDR organisms with poor response to current empirical regimens is an increasingly significant global health threat.^1^^,^^35^ Current SOC regimens, including the studied WHO recommendation (ampicillin and gentamicin) are now redundant in many areas and new regimens need to be found, or older, less commonly used antibiotics re-evaluated.^36^^,^^37^ Combination therapy, which aims to cover multiple modes of antibacterial action, needs to be further explored not only for antibacterial efficacy, but also through analysis of combined PK behaviour. For neonatal sepsis, the next step is exploring neonatal combined PK, safety and efficacy of combinations of other drugs with improved coverage against MDR pathogens.

### Conclusions

A combined model describing ampicillin and gentamicin PK through joint renal filtration clearance and an additional non-filtration term describes the observed neonatal sepsis population well. PKPD assessments suggest that good Gram-positive coverage throughout the neonatal population is provided by the regimen’s β-lactam component ampicillin. Less than one-quarter of neonates were, however, provided with efficacious coverage against Enterobacterales. When analysing a range of exemplar clinical *E. coli* strains displaying resistance to one or both agents, we found only a very weak trend towards synergy, with mostly additive effects according to FICI, indicating the need to consider alternative antibiotics for empirical therapy in areas where the burden of antibiotic resistance is high.

## Supplementary Material

dkab413_Supplementary_Data
