# Supplementary material for: Simultaneous pharmacokinetic/pharmacodynamic (PKPD) assessment of ampicillin and gentamicin in the treatment of neonatal sepsis
Source: J Antimicrob Chemother. 2021 Nov 23;77(2):448–56. doi: 10.1093/jac/dkab413 (PMC8809196; doi:10.1093/jac/dkab413)
Supplement: dkab413_Supplementary_Data [file dkab413_supplementary_data.docx]

## Supplementary data

## Ampicillin and Gentamicin bioanalysis

Plasma samples were centrifuged at 3,000 RPM (2,173 x g) for 5 minutes then separated and frozen (at -80°C) within 30 minutes of collection. Frozen samples were shipped to Analytical Services International Ltd., St. Georges University of London, UK. Ampicillin and gentamicin concentrations in plasma samples were assessed using Liquid Chromatography Tandem Mass Spectrometry assays. The lower limit of quantification was 0.5 mg/L for ampicillin and 0.1 mg/L for gentamicin. The linear range for ampicillin was 0.5-200 mg/L and for gentamicin 0.1-100 mg/L. Accuracy for ampicillin ranged from 87-110% (with CV% <8.9%) and 88-108% (with CV% <7.8%) for gentamicin over the linear range. The method was partially validated according to European Medicines Agency guidelines and following validation parameters were assessed: linearity, lower limit of quantification, accuracy, precision, matrix factor, short-term and autosampler stability. All assessed validation parameters remained within the acceptance criteria set by validation guideline.^1^


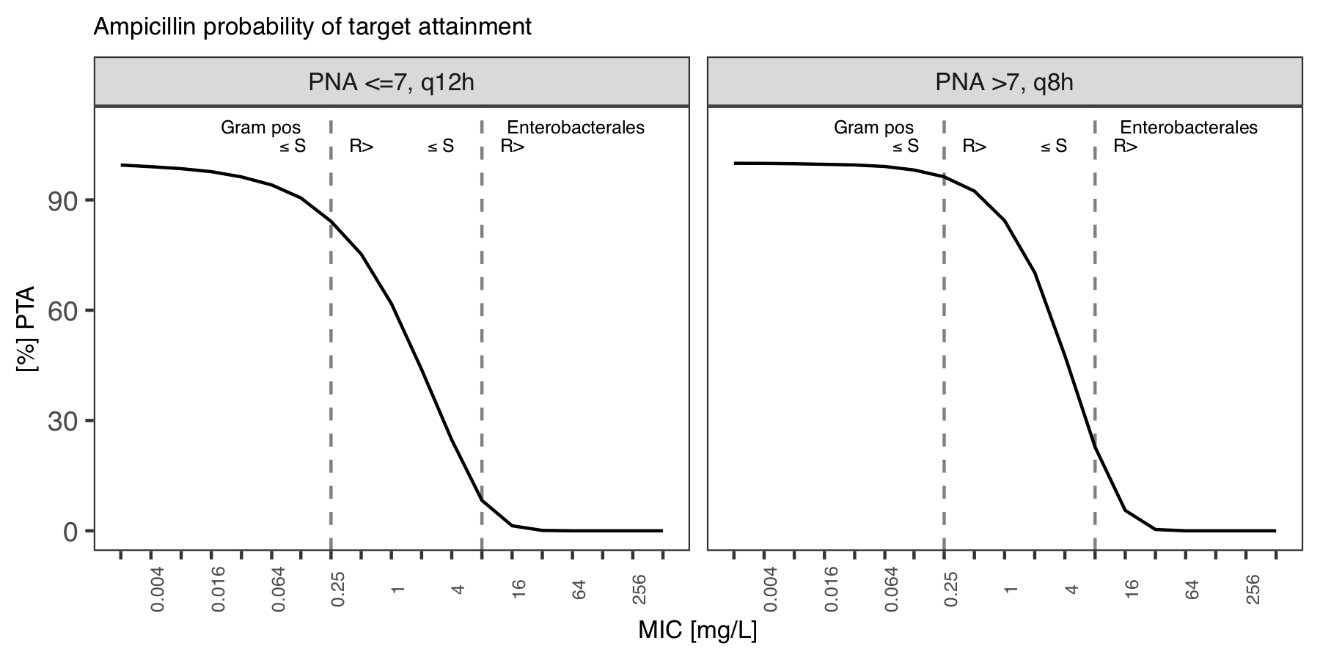


Figure S1: Ampicillin probability of target attainment (fT>MIC = 100%) for WHO pocket book dose (50mg/kg), infused over 5 min. Dashed lines, EUCAST breakpoints for *enterobacterales* and *coagulase-negative staphylococcus*.


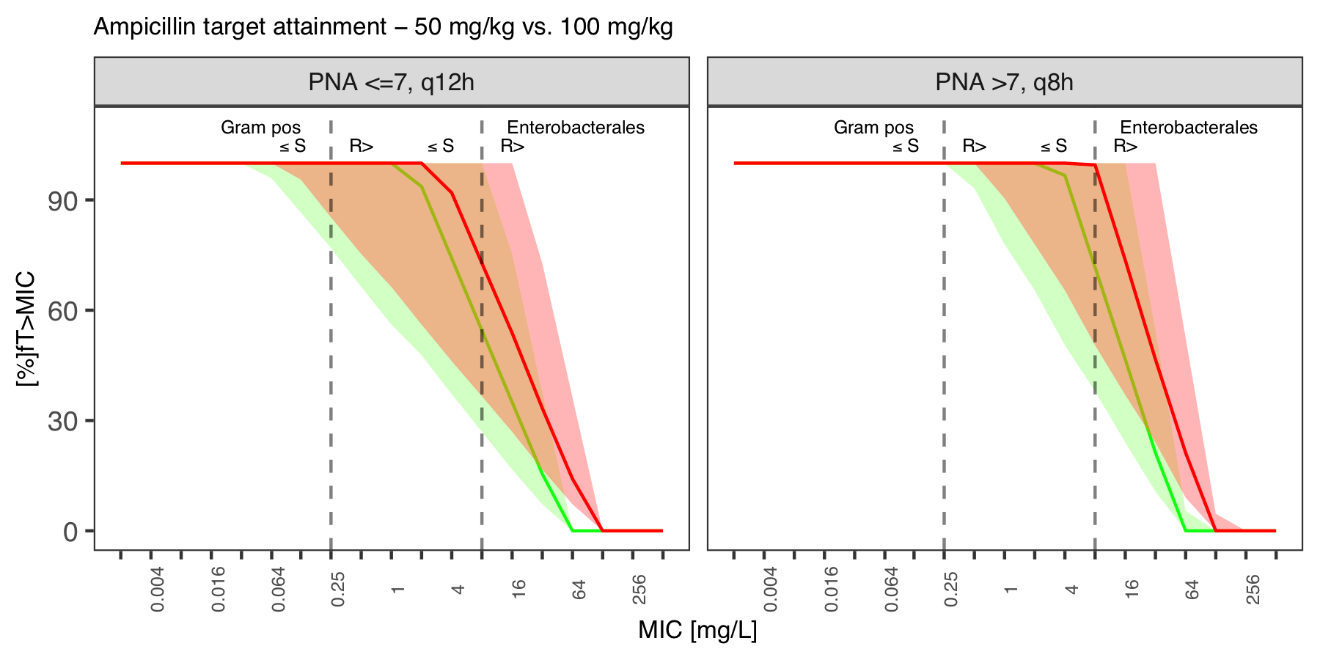


Figure S2: Target attainment as %fT>MIC against MICs for simulated ampicillin regimen. Solid line, population median; grey area, 95% prediction interval; dashed lines, EUCAST breakpoints for *enterobacterales* and *coagulase-negative staphylococcus*. Red, 100 mg/kg dose; Green, 50 mg/kg dose. Simulations with 5min injection.


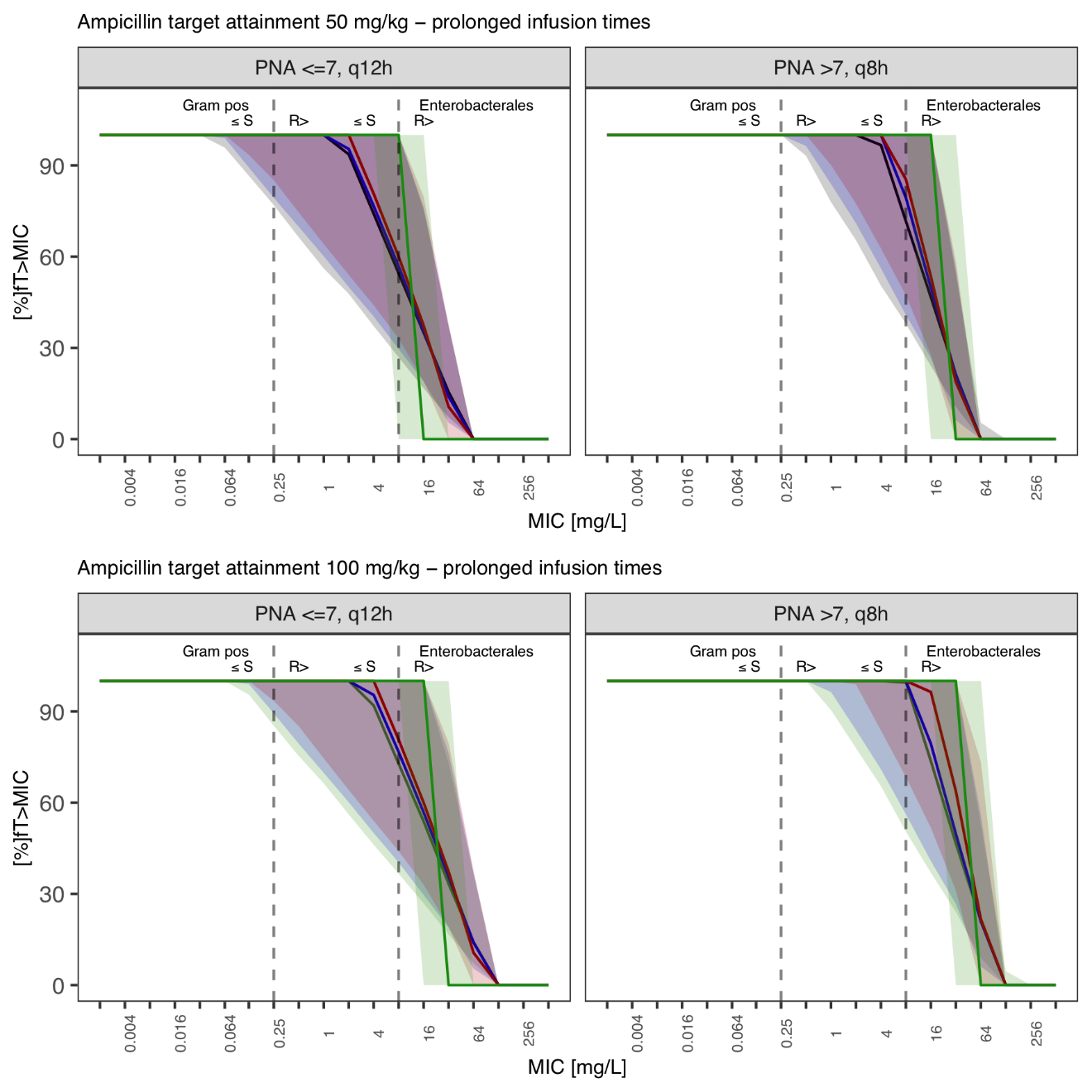


Figure S3: Target attainment as %fT>MIC against MICs for simulated ampicillin regimen. Solid line, population median; grey area, 90% prediction interval; dashed lines, EUCAST breakpoints for for *enterobacterales* and *coagulase-negative staphylococcus*. Green, continuous infusion; Red, 2hr infusion; Blue, 1hr infusion; Black, 5min injection. Top row, 50 mg/kg dose; Bottom row, 100 mg/kg dose.


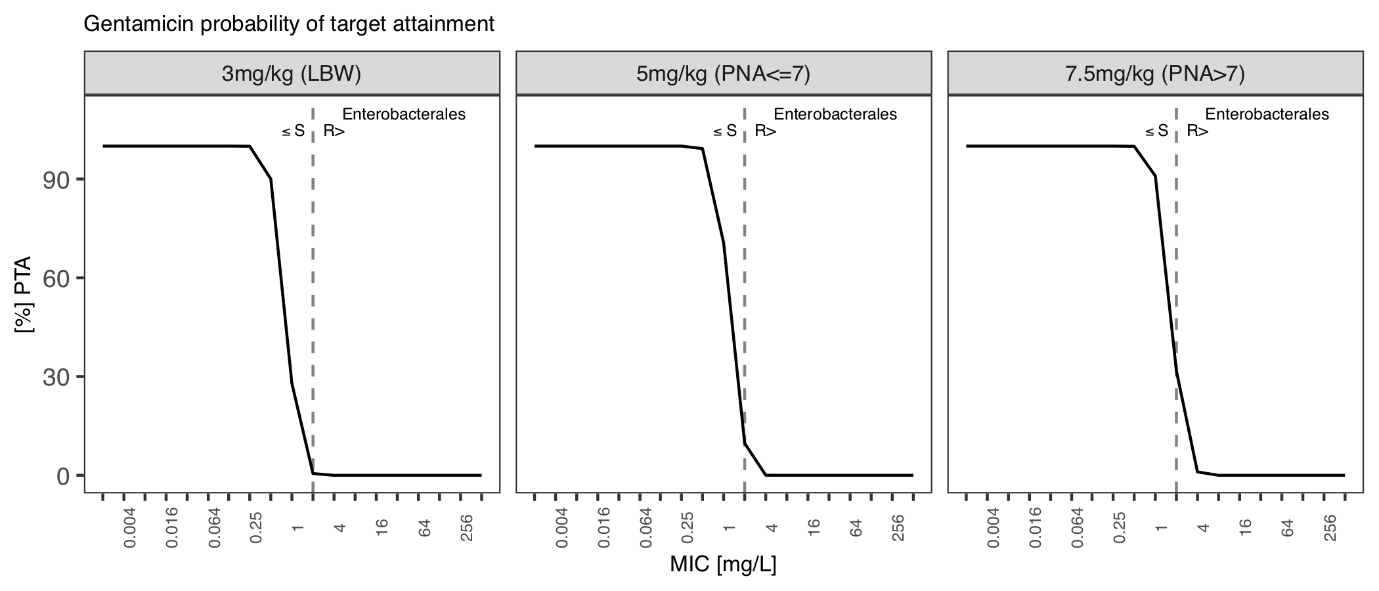


Figure S4: Gentamicin probability of target attainment (Cmax:MIC ≥ 10) at day 2 for WHO pocket book dose (3mg/kg LBW, 5mg/kg BW > 1.5kg), infused over 5 mins. Dashed line, EUCAST breakpoint for *enterobacterales*.


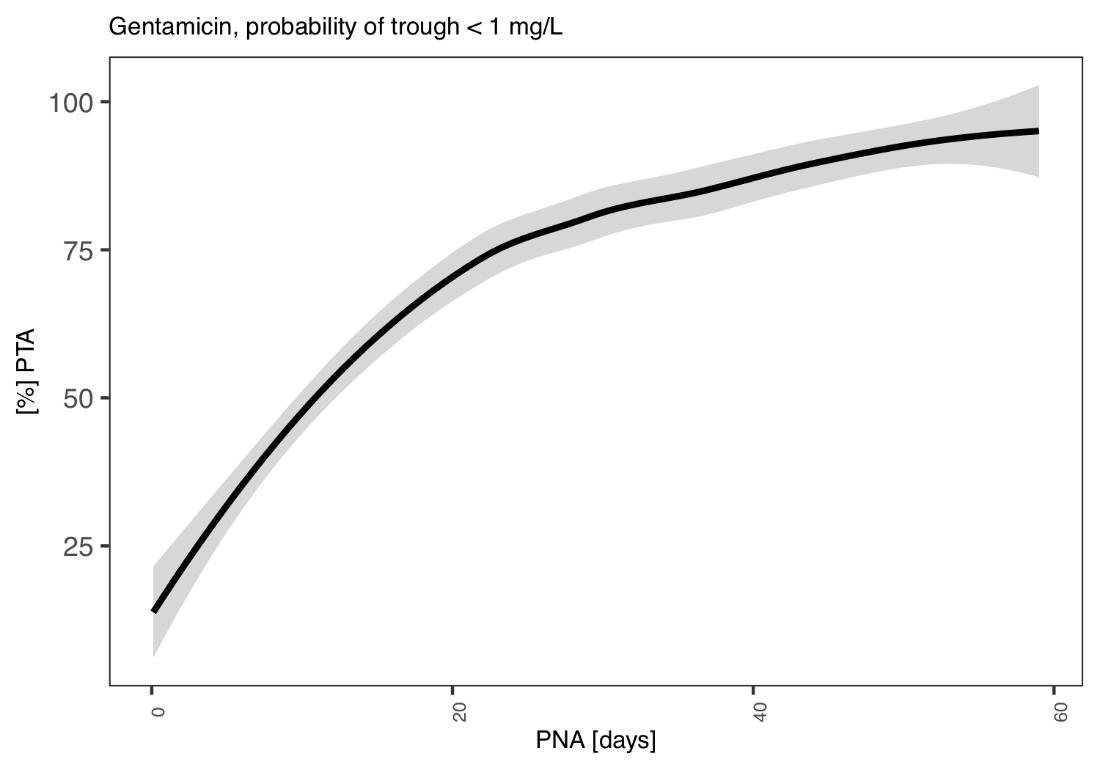


Figure S5: Probability of achieving gentamicin trough levels below 1 mg/L with the 5 mg/kg BNFc regimen. Loess fit across probability in simulated population for respective post-natal age. Black line, mean. Grey shaded area, 95% confidence interval.

References

1. European Medicines Agency – EMEA; Committee for Medicinal Products for Human Use - CHMP/EWP/192217/2009. Guideline on bioanalytical method validation. 2011.
